# Elevated plasma level of soluble triggering receptor expressed on myeloid cells-1 is associated with inflammation activity and is a potential biomarker of thrombosis in primary antiphospholipid syndrome

**DOI:** 10.1186/s13075-018-1779-5

**Published:** 2019-01-07

**Authors:** Yonatan Edel, Vitaly Kliminski, Elisheva Pokroy-Shapira, Shirly Oren, Ariela Dortort Lazar, Yael Pri-Paz Basson, Mohammad Egbaria, Yair Molad

**Affiliations:** 1 Rheumatology Unit, Rabin Medical Center – Beilinson Hospital, 4941492 Petach Tikva, Israel; 2 Laboratory of Inflammation Research, Felsenstein Medical Research Center, Rabin Medical Center – Beilinson Hospital, Petach Tikva, Israel; 3 Sackler Faculty of Medicine, Tel Aviv University, Tel Aviv, Israel

**Keywords:** Triggering receptor expressed on myeloid cells-1 (TREM-1), Thrombosis, Antiphospholipid syndrome, Biomarker

## Abstract

**Background:**

Soluble triggering receptor expressed on myeloid cells-1 (sTREM-1) is an innate-immune receptor found in blood. Its presence reflects innate immune cell activation. We sought to investigate plasma sTREM-1 levels in patients with primary antiphospholipid syndrome (PAPS).

**Methods:**

A cross-sectional, case-control design was used. Plasma sTREM-1 levels were analyzed by enzyme-linked immunosorbent assay (ELISA) in consecutive patients diagnosed with PAPS or asymptomatic antiphospholipid antibody (APLA) carriers and controls.

**Results:**

The study cohort included 33 patients with PAPS, 10 asymptomatic APLA carriers, and 73 controls. Mean plasma sTREM-1 levels were significantly higher in patients with PAPS (299.2 ± 146.7 pg/ml) and thrombotic PAPS-ever (current and past thrombotic event) (327.2 ± 151.3 pg/ml) compared with controls (230.2 ± 85.5 pg/ml; *p* = 0.006 and *p* = 0.003, respectively), patients with thrombotic PAPS compared with patients with past obstetric APS (195.12 ± 58.52 pg/ml, *p* = 0.01) and APLA carriers (215.8 ± 51.6 pg/ml, *p* = 0.02), patients with current thrombotic PAPS (429.5 ± 227.5 pg/ml) compared with patients with past thrombotic PAPS (289.5 ± 94.65 pg/ml, *p* = 0.01), and patients with PAPS who had ever had a stroke or venous thromboembolic event compared with patients who had not (*p* = 0.007 and *p* = 0.02, respectively). On receiver operator characteristic curve analysis, plasma sTREM-1 levels differentiated patients with current thrombotic PAPS from asymptomatic APLA carriers and controls, with an area under the curve of 0.7292 (*p* = 0.0014) and 0.88 (*p* < 0.0001), respectively. Multivariate regression analysis to identify sTREM-1 predictors (thrombotic PAPS-ever, age, and sex) yielded an independent association of sTREM-1 levels with thrombotic PAPS (*p* < 0.0001).

**Conclusions:**

Plasma sTREM-1 levels are significantly elevated in patients with thrombotic PAPS. Levels of sTREM-1 might serve as a biomarker for thrombosis in patients with PAPS.

## Background

Antiphospholipid syndrome (APS) is a systemic autoimmune disease characterized by thrombotic events and/or pregnancy morbidity in the presence of persistent antiphospholipid antibodies (APLA), namely lupus anticoagulant (LAC), anticardiolipin (aCL) IgG and/or IgM antibodies, and/or anti-β_2_ glycoprotein I (β_2_GPI) IgG and/or IgM antibodies [1]. The mechanism of clot formation is considered multifactorial and remains largely unknown. Although the prevalence of APLA in the general population ranges between 1% and 5%, clinically overt thrombotic APS develops in only a minority of affected individuals, suggesting that the presence of APLA alone is not sufficient to trigger clot formation, and a “second hit” with another, perhaps inflammatory, factor is required [2].

There is increasing evidence that the pathogenesis of APS involves innate immune activation, particularly via toll-like receptors (TLRs). The TLR family of pattern recognition receptors plays a pivotal role in infectious and autoimmune diseases [3]. TLR-4 is a cell-surface receptor for bacterial lipopolysaccharide (LPS) that is mainly expressed in the cells of the innate immune system. Studies have shown that β_2_GPI binds to endothelial cells through TLR-4, among other receptors [3, 4], and interacts with LPS. These findings support the hypothesis that the LPS/β_2_GPI complex, by activating signaling pathways in monocytes similar to the action of LPS, may account for TLR-4 involvement in the pathogenesis of thrombosis in APS [5, 6]. Several in-vivo studies using animal models of APS have shown that thrombus size was smaller in the LPS-nonresponsive mice (LPS^−/−^) than the LPS-responsive mice [7]. Additionally, a recent study of patients with primary APS (PAPS) reported an increase in the level of expression of TLR-2 and TLR-4 mRNA in peripheral blood mononuclear cells in association with evidence of endothelial dysfunction, arterial stiffening, and hypertrophy [8].

Triggering receptor expressed on myeloid cells-1 (TREM-1) is a recently identified DAP-12-associated cell-surface receptor expressed mainly on monocytes and neutrophils. It is involved in the amplification of TLR-4-mediated inflammatory responses [9, 10]. The expression of TREM-1 is upregulated in response to LPS, and its colligation with TLR-4 results in greater production of proinflammatory cytokines and chemokines than induced by either TREM-1- or TLR-4-mediated activation alone [9–12]. At the same time, the soluble form of TREM-1 (sTREM-1) is released and apparently exerts an anti-inflammatory effect, as indicated by findings of its inverse correlation with tumor necrosis factor-α and interleukin-1β in murine sepsis [13]. Taken together, these studies suggest that TREM-1 upregulation is associated with and amplifies TLR-4-mediated monocyte/macrophage activation and that the blood sTREM-1 level correlates with monocyte/macrophage membrane TREM-1 upregulation and innate immune cell activation.

In light of the growing evidence of the role of innate immunity, and TLR-4 in particular, in the pathogenesis of thrombosis in APS, we sought to investigate the plasma level of sTREM-1 in a cohort of patients with PAPS, the clinical association of sTREM-1 with thrombotic PAPS, and the possible use of sTREM-1 as a biomarker of thrombotic events in PAPS.

## Methods

### Study population

A cross-sectional, case-control study design was used. The cohort consisted of consecutive patients diagnosed with PAPS or asymptomatic carriers of persistently positive APLA under routine follow-up at the Rheumatology Clinic of a tertiary university-affiliated medical center. The study protocol was reviewed and approved by the local Institutional Review Board, and all participants provided written informed consent.

The diagnosis of PAPS was based on the combined presence of one positive clinical criterion and one positive laboratory criterion (aCL and/or aβ2G1 antibodies by enzyme-linked immunosorbent assay (ELISA) and/or LAC determined according to the International Society of Thrombosis and Haemostasis) on two or more occasions not less than 12 weeks apart, as stipulated in the Sydney revised Sapporo guidelines [14]. Vascular thrombosis was defined as one or more clinical episodes of arterial, venous, or small-vessel thrombosis in any tissue or organ, confirmed by objective validated criteria (unequivocal findings on appropriate imaging studies or histopathology). Pregnancy morbidity was defined as follows: one or more unexplained deaths of a morphologically normal fetus at or beyond week 10 of gestation, with normal fetal morphology documented by ultrasound or by direct examination of the fetus (intrauterine fetal death (IUFD)); or one or more premature births of a morphologically normal neonate before the 34th week of gestation because of eclampsia or severe preeclampsia defined according to standard definitions or because of recognized features of placental insufficiency (intrauterine growth restriction); or three or more unexplained consecutive spontaneous abortions before the 10th week of gestation, excluding maternal anatomic or hormonal abnormalities and paternal and maternal chromosomal causes. For the classification of PAPS, APLA positivity was defined as a moderate-to-high titer of aCL IgG/IgM antibody (> 40 GPL/MPL U/ml) and/or anti-β_2_GPI IgG/IgM above the 99th percentile (IgG/IgM aCL/anti-β_2_GPI ≥ 20 GPL/MPL U/ml) and/or positive LAC by either dilute Russell viper venom time test (> 1.4) and/or silica clotting time test (> 1.3). Patients with systemic autoimmune or rheumatic diseases, including but not limited to systemic lupus erythematosus, rheumatoid arthritis, and Sjögren’s syndrome, were excluded from the study as were patients with evidence of infectious disease within 3 months prior to blood sampling and/or evidence of active malignancy, in addition to pregnant women, women within 12 weeks after delivery or abortion, and patients with thrombosis attributed to a non-APLA cause. Asymptomatic APLA carriers were defined as consecutive patients with persistently positive aCL IgG and/or IgM and/or anti-β_2_GPI IgG and/or IgM and/or positive LAC test on two consecutive occasions at least 12 weeks apart with no evidence of thrombosis and/or obstetric features of APS and/or inflammatory or autoimmune systemic disorder, infection, malignancy and/or pregnancy or 12 weeks or less postpartum. Healthy control subjects were recruited from the hospital staff. Exclusion criteria for the control group were history of thrombosis, systemic autoimmune or inflammatory disease, current infection, current or previous malignancy, and/or pregnancy or 12 weeks or less postpartum.

### Clinical and laboratory data collection

At the study encounter, participating patients were interviewed, and demographic and clinical information was systematically collected, including current age, sex, time of diagnosis, and thrombotic and/or obstetric manifestations of PAPS. Medications used at the time of the study or within the previous 3 months were recorded, specifically low-dose aspirin (75–100 mg/day), hydroxychloroquine (200–400 mg/day), warfarin (Coumadin), new oral anticoagulants, prednisone, and statins. In addition, the individual electronic charts and the hospital’s computerized database were systematically reviewed for the following comorbidities: arterial hypertension, defined as systolic blood pressure higher than 140 mmHg and diastolic pressure higher than 90 mmHg that required antihypertensive medications; diabetes mellitus, defined as hyperglycemia requiring oral drugs and/or insulin; dyslipidemia, defined as serum total cholesterol above 200 mg/dl and/or triglycerides above 150 mg/dl and/or anti-lipidemic drug use; ischemic heart disease, defined as evidence of myocardial infarction and/or evidence of coronary artery atherosclerosis obtained by either coronary catheterization or a percutaneous coronary intervention procedure; and stroke defined by evidence of neurological deficit and/or computed tomography or magnetic resonance imaging signs of brain infarct. Estimated glomerular filtration rate (eGFR) at the time of the study encounter was calculated using the Modification of Diet in Renal Disease equation [15].

Blood was collected from all patients at the study encounter and assayed in the hospital’s routine clinical laboratories for complete blood count, erythrocyte sedimentation rate (ESR), and levels of serum high-sensitive C-reactive protein (hsCRP), ferritin, and creatinine.

The APLA results used for the purpose of the study were serum aCL and anti-β_2_GPI IgA/IgM antibodies and LAC. All factors were tested routinely at the hospital’s laboratory at the most recent clinic visit prior to the study or at the study encounter. A commercial ELISA kit was used according to the manufacturer’s instructions to analyze aCL IgG and IgM antibodies (Aesku Diagnostics GmbH&Co. KG, Wendelsheim, Germany) and anti-β_2_GPI IgG and IgM antibodies (Inova Diagnostics, Quanta Lite, San Diego, CA, USA), and LAC was assayed according to published guidelines [16].

### Measurement of plasma sTREM-1 levels

All blood samples were centrifuged at 1500 g for 15 min immediately after venous blood withdrawal and stored at −70 °C until assayed. The plasma level of sTREM-1 was analyzed using a commercial ELISA kit (Human TREM-1 DuoSet ELISA kit, DY1278B, Bio-Techne, Minneapolis, MN, USA) according to the manufacturer’s instructions [17]. The assay plates (CI9018, Corning Inc., Corning, NY, USA) were coated with the capture antibody overnight at 4 °C and then washed with 0.05% Tween®20 in phosphate-buffered saline (PBS) (PeproTech Inc., Rocky Hill, NJ, USA) and blocked with the assay reagent diluent (5% Tween®20 in PBS) for 2 h at room temperature, and then washed. Next, 100 μl of serially diluted standards (3000 ± 23.45 pg/ml), patient, and control plasma were added and incubated overnight at 4 °C. On the following day, the plates were equilibrated at room temperature for 2 h and then washed and incubated with a detection antibody diluted with the reaction diluent containing 2% heat-inactivated normal goat serum for 2 h at room temperature. Next, the plates were washed, and incubated for 20 min at room temperature with horseradish peroxidase-conjugated streptavidin diluted with the reaction diluent (1:200). The plates were washed and incubated with TMB substrate (PeproTech Inc., Rocky Hill, NJ, USA) for 20 min and the reaction was stopped with 1 N HCl (PeproTech Inc., Rocky Hill, NJ, USA). The absorbance was measured by a spectrophotometer (BioTek, Winooski, VT, USA) set at 450 nm with the correction wavelength set at 570 nm. The results were analyzed by creating a four-parameter logistic (4-PL) curve against the optical density of the standards (3000 ± 23.45 pg/ml) following the subtraction of the optical density of the averaged blank controls. All plasma samples and standards were assayed in duplicates. The difference between ELISA plates was monitored by including specific healthy control plasma samples in each assay.

### Statistical analysis

The statistical analysis was generated using SAS Software, version 9.4. Continuous variables are presented as mean and standard deviation, and categorical variables as number and percentage. Values of continuous variables were compared among three groups using a general linear model (GLM) with Tukey adjustment for multiple comparisons, and between two groups using *t* tests; categorical values were compared using the chi-square test. Correlations (*r*) were calculated by Pearson correlation. Logistic regression was used for receiver operating characteristic (ROC) curve analysis. Two-sided *p* values less than 0.05 were considered statistically significant.

## Results

### Patient characteristics

Plasma samples of 116 consecutively enrolled subjects were collected and analyzed for levels of sTREM-1. The study population consisted of 33 patients with PAPS (mean age 47.8 years, range 19–88; 77.7% women), 10 asymptomatic persistent positive APLA carriers (mean age 50.6 years, range 28–75; 90% women), and 73 healthy control subjects (42.65 years, range 18–67; 54.8% women). The demographic, clinical, and laboratory features of the three groups are shown in Table 1. Within the PAPS group, 26 patients (78.78%) had previous thrombotic (*n* = 19) or obstetric (*n* = 7) events (past PAPS). Seven patients (21.21%) were evaluated for plasma sTREM-1 level at the time of an acute thrombotic event (current thrombotic PAPS). The prevalence rates of the various types of past thrombotic events in the thrombotic PAPS group (*n* = 26) were as follows: 18 patients (69.2%) had an arterial thrombotic event, 4 patients (15.4%) had a venous thromboembolic event, and 4 patients (15.4%) had arterial and venous thromboembolic events.

**Table 1:** Demographics, clinical characteristics, and laboratory profiles of patients with PAPS and asymptomatic APLA

Parameter	PAPS *n* = 33	Asymptomatic APLA *n* = 10	Control *n* = 73	*P* value*
Female	24 (72.7%)	9 (90%)	40 (54%)	0.012
Age (years), mean (range)	47.8 (19–88)	50.6 (28–75)	42.65 (18–67)	NS
Venous thromboembolism	8 (24.24%)	0	0	–
Arterial thrombosis	18 (54.54%)	0	0	–
Recurrent fetal loss	5 (15.15%)	0	0	–
PET	4 (12.12%)	0	0	–
Intrauterine growth restriction	4 (12.12%)	0	0	–
Myocardial infarction	8 (24.24%)	0	0	–
Stroke	14 (42.42%)	0	0	–
Hypertension	11 (33.33%)	1 (10%)	0	< 0.001
Diabetes mellitus	3 (9.09%)	0	0	0.03
Dyslipidemia	11 (33.33%)	2 (20%)	0	< 0.001
ANA (mean)	1:160	1:160	NA	NS
ACL IgG (GPL-U/ml)	65.35 ± 65.36	42.68 ± 67.75	NA	NS
ACL IgM (MPL-U/ml)	36.1 ± 26.51	36.28 ± 58.59	NA	NS
β_2_GPI IgG (U/ml)	58.82 ± 67.3	34.34 ± 59.24	NA	NS
β_2_GPI IgM (U/ml)	69.3 ± 54.42	54.24 ± 68.66	NA	NS
LAC dRVVT ratio	1.59 ± 1.3	1.93 ± 1.08	NA	NS
LAC SCT ratio	1.98 ± 1.54	1.77 ± 1.34	NA	NS
Single-APLA positivity (%)	21.21%	40%	–	NS
Double-APLA positivity (%)	39.39%	0%	–	NS
Triple-APLA positivity (%)	39.39%	60%	–	NS
eGFR (ml/min/1.73 m^2^)	89.9 ± 26.4	88.99 ± 28.7	97.6 ± 48.6	0.04
hsCRP (mg/dl)	0.799 ± 1.27	0.1871 ± 0.22	NA	0.02
ESR (mm/h)	28 ± 19.6	24 ± 14.22	NA	0.009
Ferritin (mg/dl)	51.2 ± 40.75	40.65 ± 23.75	NA	0.004
Platelets (K/μl)	246 ± 75.41	243 ± 58.97	NA	NS

Values are presented as mean ± SD or *n* (%), unless otherwise stated

*ACL* anticardiolipin antibody (cutoff values for IgG 18 GPL U/ml, IgM 12 MPL U/ml), *ANA* antinuclear antibody (assayed by immunofluorescence, positive ≥ 1:80), *APLA* antiphospholipid antibodies, *β*_*2*_*GPI* anti-β_2_ glycoprotein antibody (cut-off value for IgG 20 GPL U/ml, IgM 20 MPL U/ml), *dRVVT* dilute Russell viper venom time test (negative < 1.4), *eGFR* estimated glomerular filtration rate, *ESR* erythrocyte sedimentation rate, *hsCRP* high-sensitive C-reactive protein (normal < 0.5 mg/dl), *LAC* positive lupus anticoagulant, *NA* not assayed, *NS* not significant, *PAPS* primary antiphospholipid syndrome, *PET* preeclampsia/toxemia of pregnancy, *SCT* silica clotting time test (negative < 1.3)

*P values for categorical variables were calculated with a Chi-square test, for continuous variables using a general linear model with Tukey adjustment between multiple comparisons and *t* test between two groups

### Plasma sTREM-1 levels in patients with PAPS

**Fig. 1:**
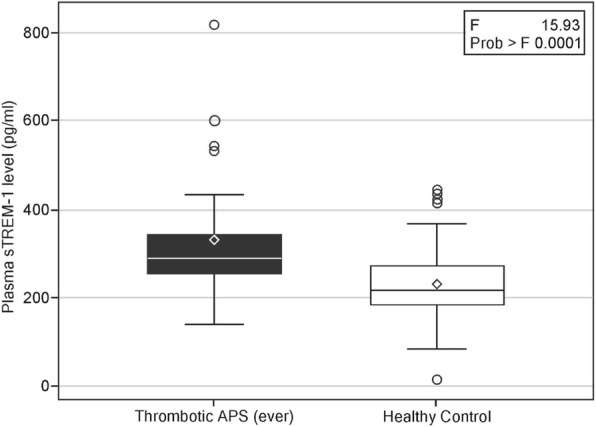
Levels of plasma soluble triggering receptor expressed on myeloid cells-1 (sTREM-1) in patients with thrombotic primary antiphospholipid syndrome (PAPS)-ever (current and past thrombotic event) (*n* = 26) compared with healthy controls (*n* = 73) (*p* = 0.0003). Data are shown as box plots. Each box represents the 25th to 75th percentiles. Lines inside the boxes represent the median. Whiskers indicate the observations directly above the lower fence or below the lower fence, where the fence is defined as 1.5 × interquartile range

**Fig. 2:**
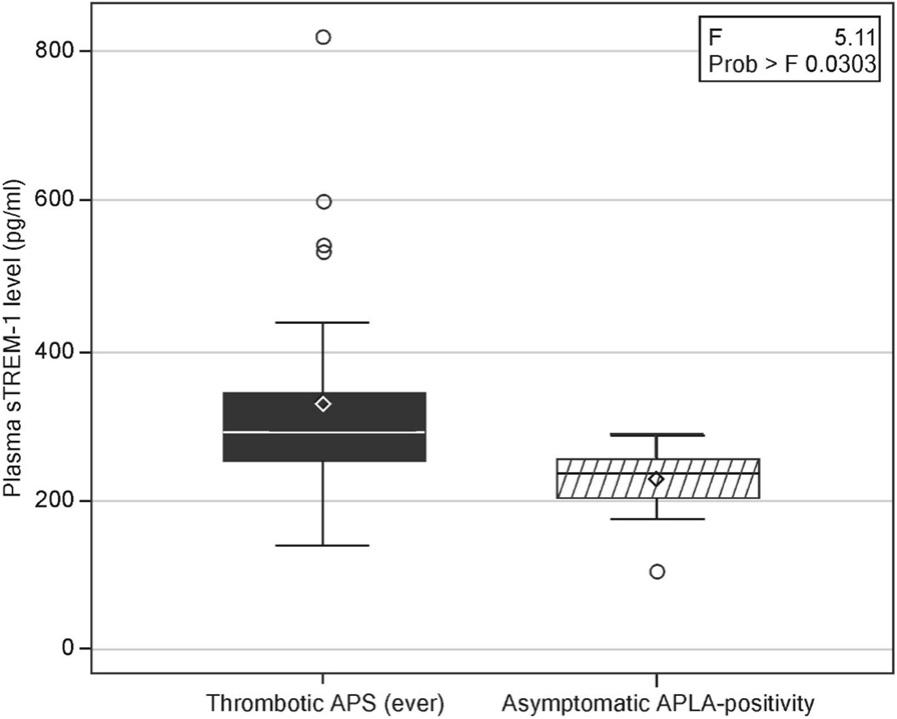
Levels of plasma soluble triggering receptor expressed on myeloid cells-1 (sTREM-1) in patients with thrombotic primary antiphospholipid syndrome (PAPS)-ever (current and past thrombotic event) (*n* = 26) compared with asymptomatic antiphospholipid antibody (APLA) carriers (*n* = 10) (*p* = 0.02). Data are shown as box plots. Each box represents the 25th to 75th percentiles. Lines inside the boxes represent the median. Whiskers indicate the observations directly above the lower fence, or below the lower fence, where the fence is defined as 1.5 × interquartile range

Analysis of plasma sTREM-1 levels by group yielded a significantly higher level in the patients with PAPS (299.2 ± 146.7 pg/ml) compared with the healthy control group (230.2 ± 85.5 pg/ml, *p* = 0.006), in the patients with thrombotic PAPS-ever (327.2 ± 151.3 pg/ml) compared with the control group (*p* = 0.0003, Fig. 1), in the patients with thrombotic PAPS compared with the patients with obstetric PAPS-past event (195.1 ± 58.5 pg/ml, *p* = 0.01), and in the patients with thrombotic PAPS compared with the asymptomatic APLA carriers (215.8 ± 51.6 pg/ml, *p* = 0.02, Fig. 2).

The plasma sTREM-1 level was positively correlated with antinuclear antibody titer (*r* = 0.33, *p* = 0.03). However, there were no statistically significant differences between patients with single, double, or triple APLA positivity (PAPS and asymptomatic groups). Specific values were as follows: single-positive (*n* = 11), mean 290.2 ± 113.5 pg/ml, median 283.3 pg/ml; double-positive (*n* = 13), mean 267.01 ± 92.6 pg/ml, median 253.7 pg/ml; triple-positive (*n* = 19), mean 282.5 ± 171.7 pg/ml, median 247.06 pg/ml. Plasma sTREM-1 levels were not significantly correlated with aCL or anti-β_2_GPI antibody titers.

### Relationship of plasma sTREM-1 levels to clinical features

**Fig. 3:**
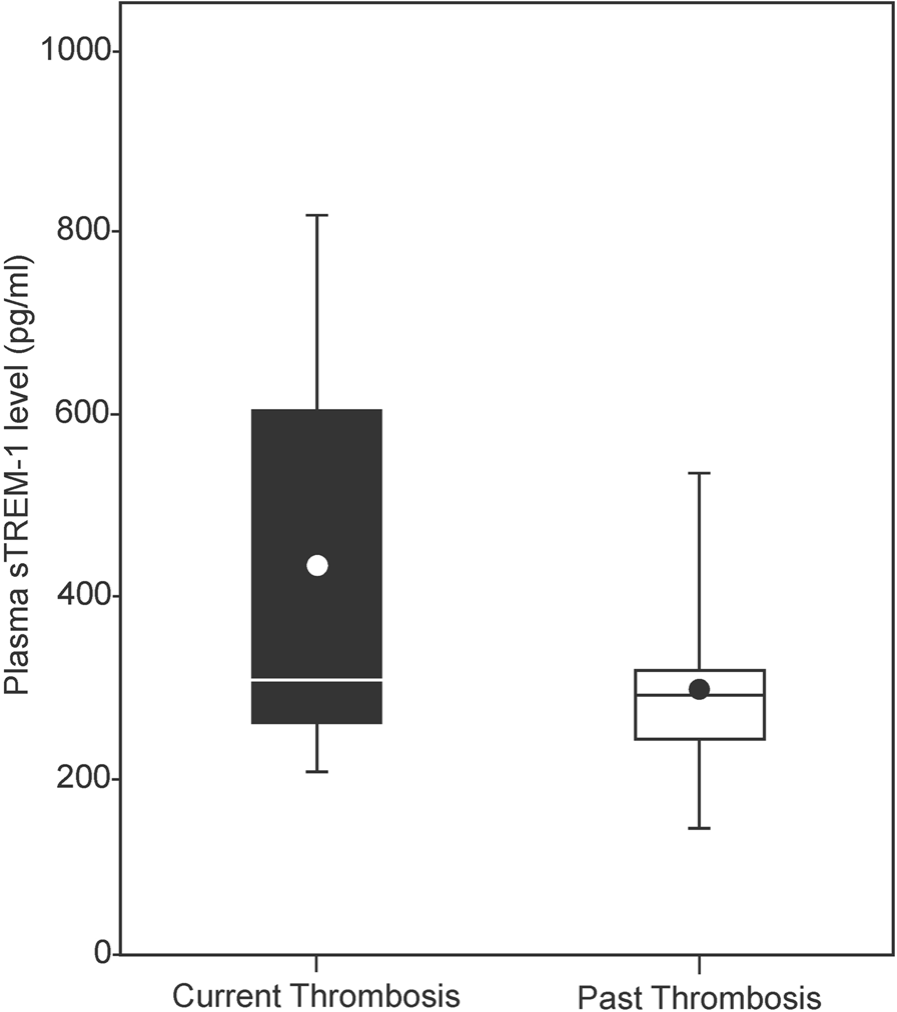
Plasma soluble triggering receptor expressed on myeloid cells-1 (sTREM-1) level in patients with thrombotic PAPS (*n* = 26): comparison of current (*n* = 19) vs. past thrombotic evet (*n* = 7) (*p* = 0.01)

Within the PAPS group, higher plasma sTREM-1 levels were positively associated with current thrombotic events (429.5 ± 227.5 pg/ml) compared with past thrombotic events (289.5 ± 94.65 pg/ml, *p* = 0.01) (Fig. 3) as well as to past obstetric events (195.1 ± 58.5 pg/ml, *p* = 0.0001). The association remained significant on comparison of the patients with thrombotic PAPS-ever with the asymptomatic APLA carrier group (215.8 ± 51.6 pg/ml, *p* = 0.0002) and the healthy controls (230.2 ± 85.5 pg/ml, *p* < 0.0001). In the patients with obstetric PAPS, there was a positive correlation of plasma sTREM-1 level with number of past pregnancies and fetal loss, but it did not reach statistical significance (*r* = 0.2, *p* = 0.06).

The plasma sTREM-1 level was significantly higher compared with controls in the patients with stroke-ever (307.2 ± 105.6 pg/ml, *p* = 0.03), myocardial infarction-ever (371.6 ± 250.04 pg/ml, *p* = 0.009), and venous thromboembolic event-ever (366.67 ± 203.79 pg/ml, *p* = 0.0012) (Table 2). Among the patients with PAPS, the plasma sTREM-1 level was significantly higher in those who had stroke-ever than in those who had not (307.2 ± 105.6 pg/ml vs. 240.5 ± 107.2 pg/ml, *p* = 0.007), and in those who had a venous thromboembolic event than in those who had not (366.7 ± 203.8 pg/ml vs. 239.8 ± 94.2 pg/ml, *p* = 0.02). There were no significant findings for myocardial infarction.

**Table 2:** Plasma levels of sTREM-1 by clinical manifestations of PAPS (*n* = 33) compared with controls (*n* = 73)

Clinical manifestation of PAPS (*n*)	Mean ± SD (pg/ml)	Median (pg/ml)	Range (pg/ml)	*P* value*
Past thrombosis (19)	289.5 ± 94.65	287.5	138.04–532.7	NS
Current thrombosis (7)	429.6 ± 227.5	303.3	203.3–817.6	< 0.0001
Past obstetric event (7)	195.1 ± 58.5	198.3	90.4–284.2	NS
Current arterial thrombosis (5)	377.2 ± 180.41	283.3	203.3–601.4	0.001
Venous thromboembolism-ever (9)	366.67 ± 203.79	316.82	141.98-817.62	0.012
MI-ever (5)	371.6 ± 250.04	257.7	238.42–817.62	0.003
Stroke-ever (14)	307.2 ± 105.6	287.9	170.02–540.29	0.03

*MI* myocardial infarction, *NS* not statistically significant, *PAPS* primary antiphospholipid syndrome, *sTREM-1* soluble receptor expressed on myeloid cells-1

*Analysis of differences in mean plasma sTREM-1 level between patients with various clinical manifestations of PAPS and healthy controls; mean sTREM-1 of the healthy control group, 230.18 ± 85.52 pg/ml

**Fig. 4:**
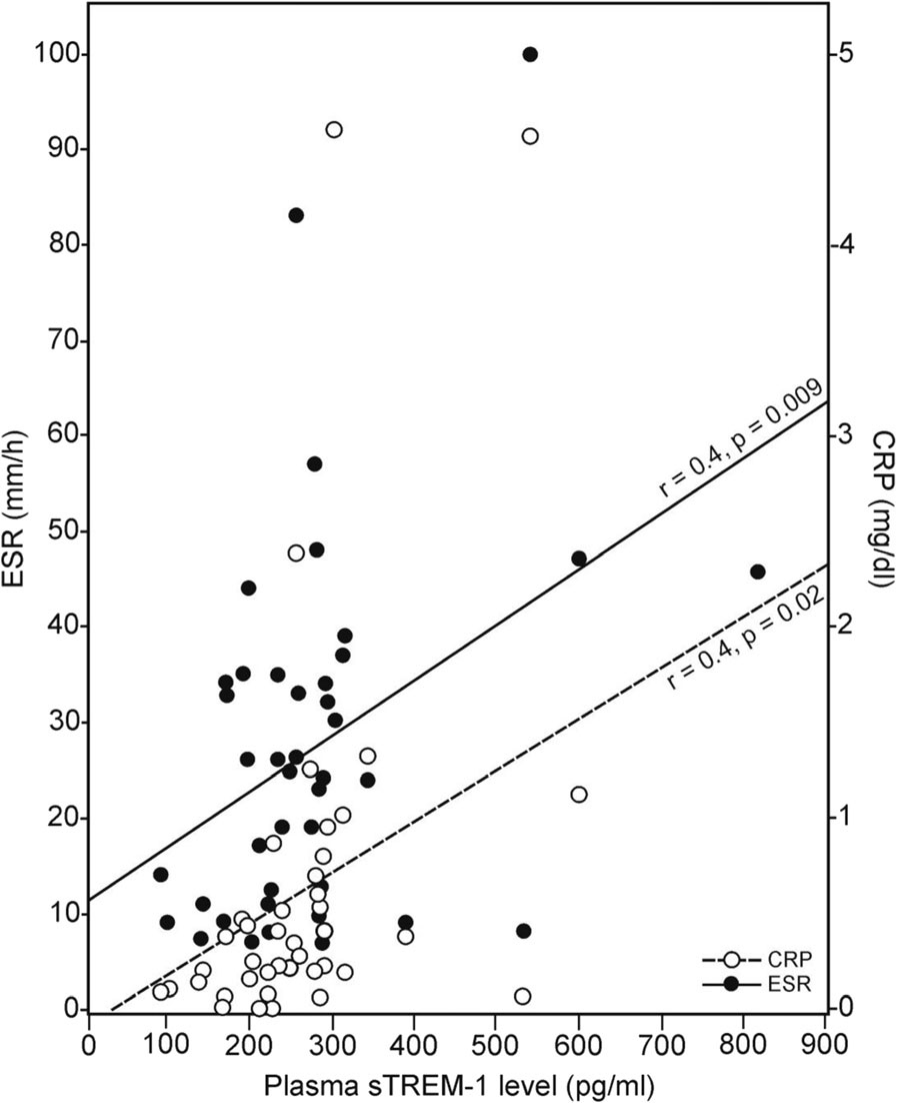
Plasma soluble triggering receptor expressed on myeloid cells-1 (sTREM-1) level correlated with elevated erythrocyte sedimentation rate (ESR; *r* = 0.4, *p* = 0.009) and elevated serum high-sensitive C-reactive protein (hsCRP) level in patients with PAPS (*r* = 0.4, *p* = 0.02)

To test whether plasma sTREM-1 level was associated with inflammatory activity in the patients with PAPS, we performed a correlational analysis of plasma sTREM-1 level with ESR and serum hsCRP levels, platelet count, and serum ferritin level, all of which are clinical biomarkers of the inflammatory acute phase response. We found a significant positive correlation for plasma sTREM-1 level with higher ESR (*r* = 0.4, *p* = 0.009) and higher hsCRP level (*r* = 0.4, *p* = 0.02) (Fig. 4).

No significant positive relationship was found between plasma sTREM-1 level and the presence of comorbidities (hypertension, diabetes mellitus, dyslipidemia, or history of a malignant disorder) or current use of anticoagulants (warfarin or new oral anticoagulants), or low-dose aspirin, hydroxychloroquine, prednisone, or statins.

The plasma sTREM-1 level was significantly associated with current age in the healthy control group (*r* = 0.63, *p* < 0.0001) but not in the PAPS or asymptomatic APLA carrier groups. Since renal function based on GFR physiologically decreases with age [18], we assumed that the age-related increase in plasma sTREM-1 level in the healthy subjects was associated with decreased renal clearance of sTREM-1. Indeed, we found a negative association for plasma sTREM-1 level and eGFR (*r* = −0.2, *p* = 0.06), but it was statistically significant only in the control group (*r* = −0.6, *p* < 0.0001). Thus, the correlation observed between plasma sTREM-1 and renal function in the control group is unrelated to levels in the patients with PAPS.

**Fig. 5:**
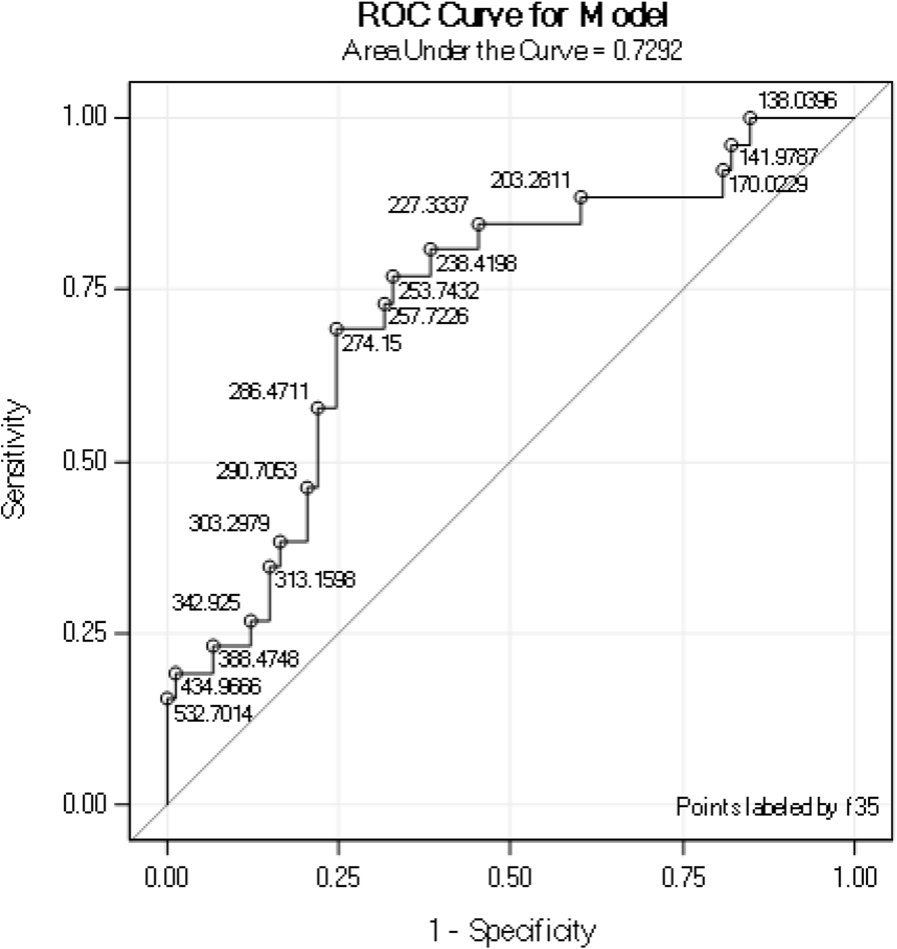
Receiver operating characteristic (ROC) curve of plasma sTREM-1 levels in patients with PAPS (*n* = 33) compared with asymptomatic persistent APLA carriers (*n* = 10) and healthy controls (*n* = 73). AUC was 0.73 (95% CI 1.003–1.013, *p* = 0.0014)

**Fig. 6:**
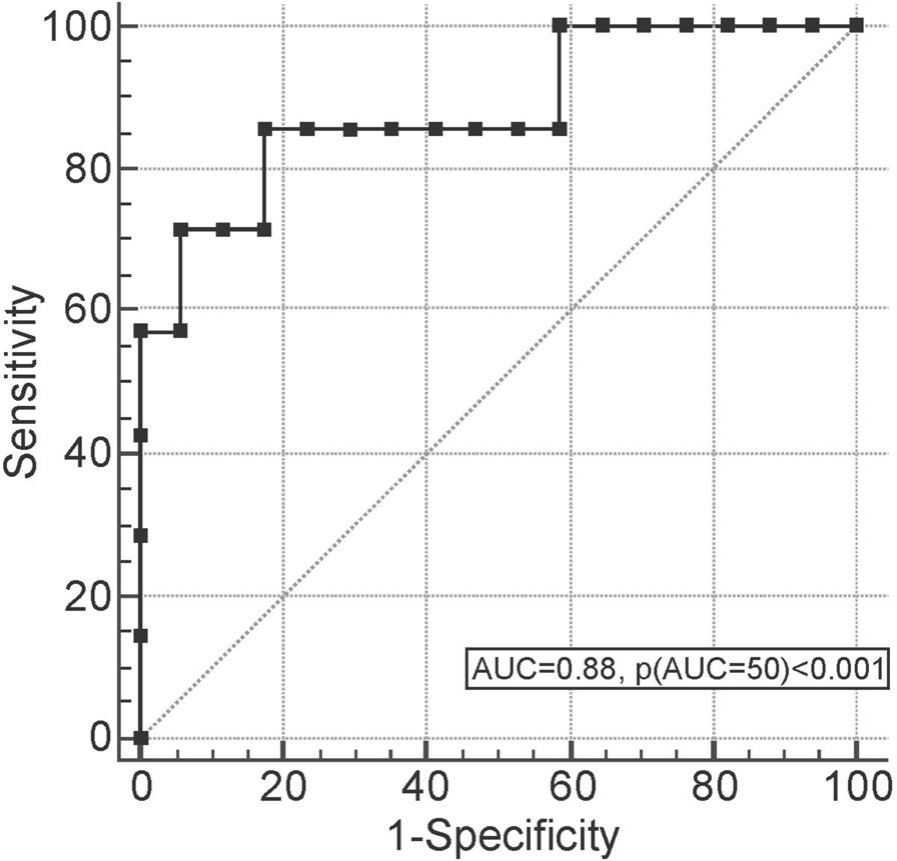
Receiver operating characteristic curve of plasma sTREM-1 levels in patients with current thrombotic PAPS (*n* = 7) compared with asymptomatic persistent APLA carriers (*n* = 10) and healthy controls (*n* = 73). Area under the curve (AUC) was 0.88 (95% CI 0.686–0.977, *p* < 0.0001)

A ROC analysis was performed to evaluate the value of plasma sTREM-1 in discriminating patients with current thrombosis from other patients with PAPS and from asymptomatic APLA carriers and healthy controls. The results showed that a cutoff plasma sTREM-1 level of 281 pg/ml had a sensitivity of 65.4% and specificity of 100% for thrombotic events-ever in the PAPS group. A cutoff of 284 pg/ml had a sensitivity of 57.1% and specificity of 100% for current thrombotic PAPS. In the subgroup of thrombotic PAPS-ever, the area under the curve (AUC) was 0.73 for plasma sTREM-1 (95% confidence interval (CI) 1.003–1.013, *p* = 0.0014; Fig. 5). No significance was found for ESR or serum ferritin level. In the subgroup of current thrombotic PAPS (*n* = 7), the AUC for plasma sTREM-1 was 0.88 (95% CI 0.686–0.977, *p* < 0.0001; Fig. 6), similar to that for the inflammatory biomarkers serum hsCRP (AUC = 0.96, 95% CI 0.788–0.999, *p* < 0.0001) and serum ferritin (AUC = 0.801, 95% CI 0.571–0.941, *p* = 0.004).

## Discussion

In this case-control study, we found significantly elevated levels of plasma sTREM-1 in patients with current thrombotic PAPS compared with past thrombotic PAPS patients as well as compared with asymptomatic persistent positive APLA carriers and healthy controls (Table 2, Figs. 1, 2, and 3). Plasma sTREM-1 levels positively correlated with thrombotic events in patients with PAPS (Table 2) as well as with high levels of the inflammatory biomarkers ESR (*r* = 0.4, *p* = 0.009) and hsCRP (*r* = 0.4, *p* = 0.02, Fig. 4), suggesting that it is associated with a low-grade inflammatory state. Interestingly, neither APLA titers nor the presence of single, double, or triple positivity was correlated with levels of plasma sTREM-1 in our cohort.

Although persistently positive triple-positive APLA is a strong risk factor for thrombosis in APS [19], the presence of APLA is by itself not sufficient to trigger clot formation [3]. Previous findings in patients with APS of elevated levels of inflammatory markers, including CRP and serum amyloid-A [20–25] relative to controls, as well as activated monocytes [26] and increased production of proinflammatory cytokines relative to controls [27, 28], suggest that PAPS is associated with a low-grade inflammatory state and an innate immune response. Inflammation involving the activation of endothelial cells, monocytes, and platelets has been shown to play a role in clot formation in animal models of APS [29–31]. Anti-β_2_GPI antibodies can trigger endothelial cell surface molecules such as annexin A2 (bound by β_2_GPI) and TLR-4 [32] and induce monocytes to increase tissue factor expression and release tumor necrosis factor α [33].

An experimental study of arterial thrombosis comparing wild-type with TLR-4-deficient mice treated with APLA [34] showed that TLR-4 modulates APLA-mediated prothrombotic effects by increasing monocyte production of tissue factor [34]. Moreover, mice treated with APLA together with a TLR-4 agonist (LPS) expressed higher levels of tissue factor activity in plasma and leukocytes [34]. Similarly, another in-vivo study found that clot size was smaller in LPS-nonresponsive mice (LPS^−/−^) than in LPS-responsive mice [7]. Accordingly, human anti-β_2_GPI antibodies derived from patients with thrombotic APS showed upregulated tissue factor expression in a TLR-4-, p38 MAP kinase-, and NF-kappaB-dependent pathway [35]. These findings are in line with the two-hit hypothesis of APS-associated thrombosis which suggests that a “first hit” injury disrupts the endothelium and a “second hit” potentiates thrombus formation [3].

TREM-1 is a newly identified member of the immunoglobulin superfamily of receptors expressed in TLR-4-mediated activated macrophages and neutrophils. Activation of the innate immune system in response to such triggers as LPS-mediated TLR-4 activation leads to degranulation, respiratory burst, release of proinflammatory cytokines, and phagocytosis [36]. During bacterial invasion, the TLR-4-induced inflammatory response is amplified [9–12, 37] by the integration of TLR-4 with activated membrane TREM-1 [38]. Studies in humans have shown that upregulation of monocyte- and neutrophil-membrane TREM-1 during endotoxemia is associated with an increased release of sTREM-1 in blood and other biological fluids [13, 39]. This process also occurs in various noninfectious, chronic inflammatory disorders [40], including rheumatoid arthritis [41, 42] and systemic lupus erythematosus [17, 43]. Thus, the plasma level of sTREM-1 may serve as a reliable biomarker for TLR-4-mediated innate immune activation in infectious as well as sterile inflammatory disorders, including autoimmune diseases.

The results of our study suggest that thrombotic PAPS is characterized by an innate immune activation state, in accordance with earlier reports of activated monocytes in patients with APS [26–28]. Indeed, APLA-induced activation of the TLR-4-dependent signaling pathway in endothelial cells [4] and monocytes [34, 44], as well as neutrophils [45], has been demonstrated in APS. A TLR-4-mediated innate immune activation along with TREM-1 upregulation is supported by the correlation found in our study between elevated plasma levels of sTREM-1 and a low-grade inflammatory state in the patients with current or past thrombotic events. Thus, TREM-1 upregulation might be involved in the TLR-4-mediated mechanism of clot formation in APS. Another potential role for TREM-1 in thrombotic APS was suggested by recent data showing that TREM-1 is constitutively expressed in platelet α-granules and which, upon platelet activation, is mobilized to the platelet surface [46]. Pharmacologic inhibition of TREM-1 in platelets from humans and also from trem-1^−/−^ mice reduced both the platelet activation as well as the platelet aggregation induced by collagen, adenosine diphosphate, and thrombin [46]. Moreover, in vivo TREM-1 inhibition decreased thrombus formation in a carotid artery model of thrombosis and protected mice during pulmonary embolism. Thus, TREM-1 may participate in platelet aggregation, a pivotal step in clot formation.

Plasma sTREM-1 levels were positively correlated with older age in the healthy control group (*r* = 0.63, *p* < 0.0001) but not in the PAPS and asymptomatic APLA carrier groups. Given the physiological decrease in glomerular filtration rate with aging [18], we assume this finding is attributable to decreased renal clearance of sTREM-1. The lack of association of the plasma sTREM-1 level with age or eGFR in the patients with PAPS and the asymptomatic APLA carriers suggests that neither age nor renal function has clinical significance with respect to plasma sTREM-1 levels in these patient groups.

Our study has several limitations. The small number of asymptomatic APLA carriers in our cohort and the cross-sectional design of the study preclude any conclusions regarding the power of plasma sTREM-1 levels to predict a thrombotic event in asymptomatic APLA carriers or patients with obstetric PAPS. These issues warrant a prospective, longitudinal study of a larger group of asymptomatic APLA carriers. Further studies are also needed to determine whether plasma sTREM-1 levels can discriminate arterial from venous thrombosis. As we excluded pregnant women with APLA, we could not determine the significance of plasma sTREM-1 in predicting the occurrence or severity of obstetrical fetal and maternal morbidity in APS.

## Conclusion

Taken together, our findings of an elevated plasma sTREM-1 level in patients with thrombotic PAPS and its correlation with elevated levels of ESR and serum hsCRP suggest that thrombotic APS is characterized by TLR-4-mediated innate immune activation [34–36] and possibly platelet activation [46]. Moreover, the correlation of elevated plasma sTREM-1 with venous and arterial thrombotic events suggests that plasma sTREM-1 can be used as a biomarker of thrombosis during follow-up of patients with PAPS.

## Abbreviations

aCL: Anticardiolipin
APLA: Antiphospholipid antibody
APS: Antiphospholipid syndrome
AUC: Area under the curve
CI: Confidence interval
eGFR: Estimated glomerular filtration rate
ESR: Erythrocyte sedimentation rate
GPI: Glycoprotein I
hsCRP: High-sensitive C-reactive protein
IUFD: Intrauterine fetal death
LAC: Lupus anticoagulant
LPS: Lipopolysaccharide
PAPS: Primary antiphospholipid syndrome
ROC: Receiver operating characteristic
sTREM-1: Soluble triggering receptor expressed on myeloid cells-1
TLR: Toll-like receptor
TREM-1: Triggering receptor expressed on myeloid cells-1
